# Plexiform Neurofibroma of the Vulva in a Patient With Neurofibromatosis Type 1

**DOI:** 10.7759/cureus.100233

**Published:** 2025-12-28

**Authors:** Salma Alkharusi, Aya A Allamki, Abir Almamari

**Affiliations:** 1 Dermatology Department, Rustaq Polyclinic, Rustaq, OMN; 2 Dermatology Department, Oman Medical Specialty Board, Muscat, OMN

**Keywords:** neurofibromatosis type 1, oman, plexiform neurofibroma, tumour, vulva

## Abstract

Plexiform neurofibroma (PNF) is a rare but pathognomonic tumour associated with neurofibromatosis type 1 (NF-1), an autosomal dominant neurocutaneous disorder. Although PNFs can affect various body regions, vulvar involvement is exceptionally uncommon. This report describes a 16-year-old female patient who presented at a dermatology center with a painless, hanging mass on the right labia majora, accompanied by multiple skin café-au-lait spots, freckling, and cutaneous neurofibromas. Clinical evaluation, genetics testing, imaging, and histopathological analysis confirmed a diagnosis of labia majora PNF. Surgical resection was performed twice due to recurrence. Given the psychosocial and functional challenges, including gait limitation, embarrassment, and psychological distress, associated with this rare presentation, a multidisciplinary approach with an individualised management plan and long-term follow-up is essential. This case emphasises the need to raise awareness of vulvar neurofibromas in NF-1 patients to ensure timely diagnosis and intervention.

## Introduction

Plexiform neurofibroma (PNF) is an irregular, painless, benign, and slowly growing tumour of the peripheral nerve sheath. It is often described as a "bag of worms" due to its infiltrative nature. Unlike other neurofibroma subtypes, PNFs can involve multiple nerve fascicles and are present in approximately 5-15% of patients with neurofibromatosis type 1 (NF-1) [1]. Vulvar involvement in PNFs is exceedingly rare. A review identified only about 31 cases of genitourinary neurofibromas reported in the literature, of which a small subset involved the vulva, particularly the labia majora [2]. Clinically, vulvar PNFs may mimic more common vulvar masses such as Bartholin's cyst, lipoma, and fibroepithelial polyp. A key concern with PNFs in such rare locations is their psychosocial distress and functional challenges, in addition to their potential for malignant transformation, necessitating repeated surgeries due to a high recurrence rate [3].

## Case presentation

**Figure 1:**
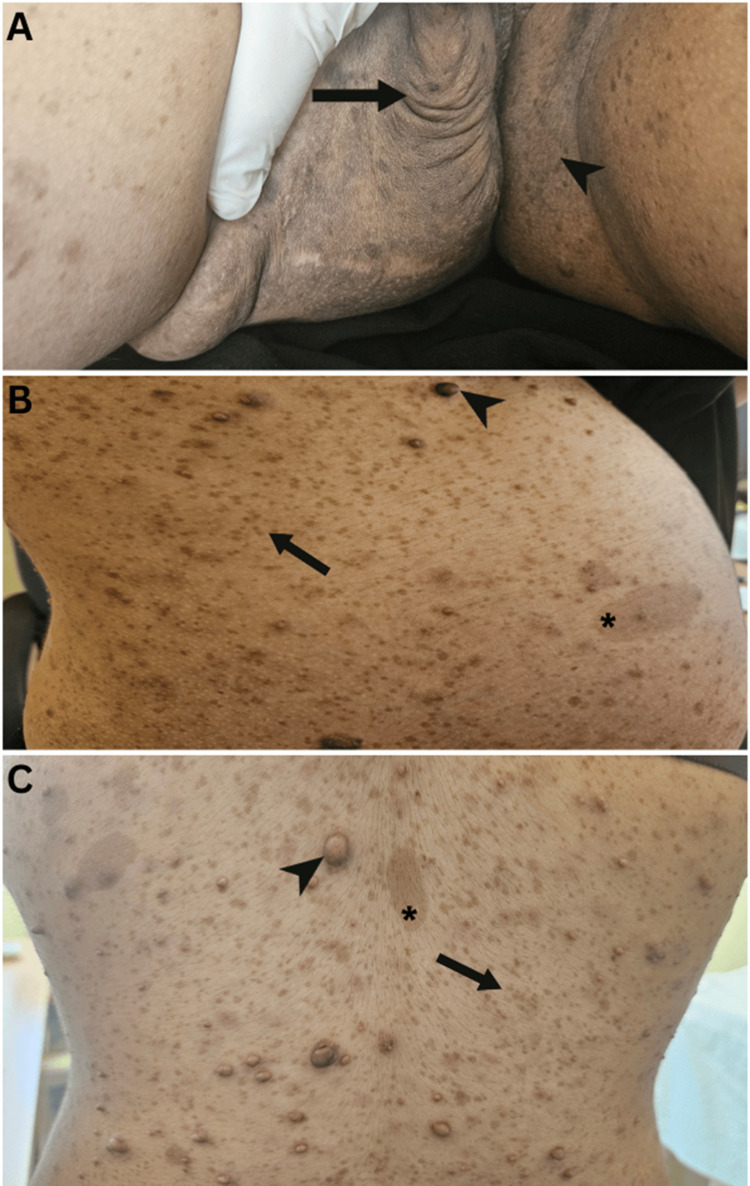
(A) Vulval swelling due to plexiform neurofibroma (arrow) and inguinal freckling (arrowhead). (B) Multiple freckles distributed over the abdomen, chest, and back (arrow), neurofibromas (arrowhead), and café-au-lait spots (asterisk). (C) Multiple freckles over the back (arrow), neurofibromas (arrowhead), and café-au-lait spots (asterisk).

A 16-year-old post-pubertal girl presented at a dermatology clinic with a progressively growing, painless, right-sided vulvar swelling that had been present since early childhood. The lesion had gradually increased in size, eventually causing difficulty in walking, which prompted her to seek medical attention. There was no history of similar swellings, trauma, or any other significant past medical conditions. Her father had similar café-au-lait spots but no tumours, and the other family history was unremarkable. On examination, a soft, compressable mobile skin-colored ill-defined subcutaneous mass with no surface changes, measuring 7 × 8 cm in diameter, was noted at the right labium majus, attached to the overlying skin without the involvement of the labia minora or clitoris. Additional findings included multiple freckles over the face, limbs, trunk, and both axillae since birth which increased in number and size, multiple café-au-lait spots (>6), and numerous cutaneous neurofibromas over the trunk (Figure 1), meeting the core NIH diagnostic criteria of NF-1.

There was no evidence of inguinal lymphadenopathy, hepatosplenomegaly, or other systemic abnormalities.

**Figure 2:**
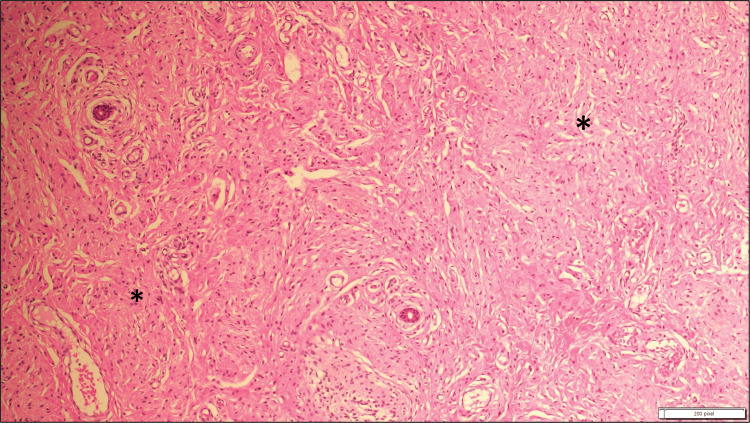
Haematoxylin and eosin-stained spindle cells (asterisks) exhibit elongated, wavy nuclei with minimal cytologic atypia, and the background stroma appears myxoid. No evidence of mitotic activity is seen (at ×10 magnification).

**Figure 3:**
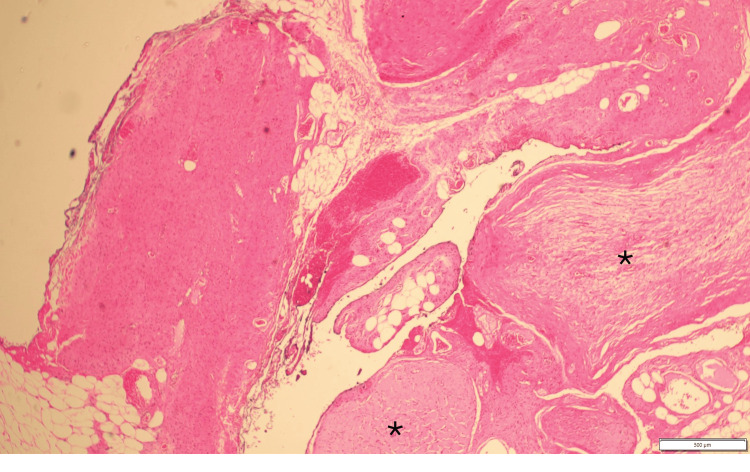
This view shows the hallmark plexiform architecture (asterisks) with nerve bundles arranged in a tortuous, intertwining pattern resembling a "bag of worms." These bundles are composed of spindle cells with elongated, wavy nuclei and are embedded in a myxoid stroma (haematoxylin and eosin staining at ×10 magnification).

Magnetic resonance imaging (MRI) revealed a 7.6 × 7.3 × 1.2 cm mass in the right labium majus. Given the size and progressive nature of the lesion, the patient was referred to surgery for an excisional biopsy. The skin biopsy showed dermal infiltration by spindle cell proliferation, permeating fat diffusely and surrounding skin adnexal structures. Histopathological analysis identified Wagner-Meissner bodies and a focal plexiform pattern, confirming the diagnosis of PNF (Figure 2, Figure 3). The patient was referred to ophthalmology, to rule out NF-1-related eye manifestations such as optic pathway gliomas and Lisch nodules, and no eye abnormalities were detected.

**Figure 4:**
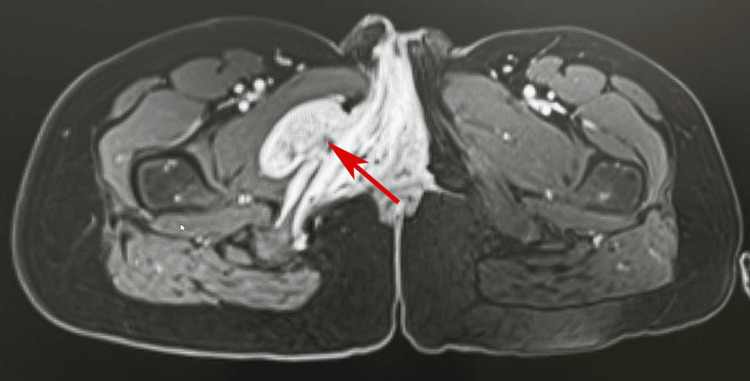
Magnetic resonance imaging with contrast shows unilateral right vulval enlargement containing a well-defined large mass measuring 7.3 × 5.3 × 1.6 cm (arrow).

She underwent surgical resection in India, and she was counselled regarding the risk of recurrence and scheduled for regular follow-up. Three years after the surgery, she had a recurrence at the same site of previous PNF, and MRI revealed a well-defined right unilateral vulvar mass measuring 7.3 × 5.3 × 1.6 cm (Figure 4).

Therefore, the patient came back and was referred to plastic surgery for further management. She remains under regular surveillance to monitor disease progression.

## Discussion

NF-1 is an autosomal dominant neurocutaneous disorder caused by a mutation in the NF-1 gene located on chromosome 17. This gene encodes neurofibromin, a tumour suppressor protein involved in regulating cell growth. NF-1 is a multisystemic disorder, affecting the peripheral nerves, bones, skin, and, in rare cases, female urogenital tract [4].

PNFs are uncommon but pathognomonic tumours for NF-1, originating from neural crest cells. PNFs affect approximately 5-15% of patients in a clinically apparent form, although radiological studies suggest that subclinical lesions may be present in up to 30-50% [5]. These tumours present as multiple discrete growths arising from a nerve or multiple nerve branches and plexuses. PNFs typically develop during early childhood and exhibit rapid growth, particularly during adolescence, puberty, and pregnancy [6]. While PNFs can involve the cranial nerves and deeper tissues without evidence of superficial growth [7], isolated cases have been reported in the palms, tip of the nose, orbit, oropharynx, and tongue [8].

External genital involvement in NF-1 is rare, with vulvar PNFs being the most frequently reported manifestation in affected females. Clitoromegaly is the most common presentation, while PNF involving the labia majora without clitoromegaly, as seen in the current case, is exceptionally rare [9,10]. The differential diagnoses for vulvar masses include the following: lipoma, lipoblastoma, angiomyofibroblastoma, aggressive angiomyxoma, cellular angiofibroma, nevus lipomatosus cutaneous superficialis, and other neurogenic tumours such as schwannoma [11].

PNFs have also been reported in the endometrium, myometrium, ovaries, vagina, and urinary tract, although these presentations remain infrequent. In males, genital involvement is uncommon, with penile enlargement being the most frequently documented presentation [3].

Approximately 8-15% of patients with PNFs experienced malignant transformation into neurofibrosarcomas. Additionally, compression of surrounding tissues can lead to significant morbidity, deformity, and functional impairment, necessitating careful monitoring and timely intervention [12].

Surgical resection remains the primary treatment modality; however, complete excision is often challenging due to the diffuse nature of PNFs, their involvement of multiple nerve structures, and infiltration into surrounding tissues. Recurrence following surgery is common, and lesions that cannot be completely resected should be monitored closely for functional deterioration and potential malignant transformation [6,13].

Targeted suppression of the RAS/mitogen-activated protein kinase (MAPK) pathway has propelled recent advancements in the treatment of PNFs in NF-1, with MEK inhibitors emerging as the first disease-modifying systemic therapies. Selumetinib was approved by the Food and Drug Administration (FDA) in 2020 for children aged ≥2 years. More recently, mirdametinib received FDA approval in 2025 for both pediatric and adult patients with symptomatic, unresectable PNFs [14].

## Conclusions

PNFs are rare tumours associated with NF-1, presenting significant diagnostic and therapeutic challenges in such atypical sites of involvement due to their infiltrative nature, functional and psychological burden, and risk of recurrence. While surgical excision remains the primary treatment, achieving complete resection is often difficult, and recurrence rates are high. A multidisciplinary approach involving regular monitoring, functional assessment, and psychological counselling is crucial in optimising patient outcomes. Early diagnosis and long-term follow-up are essential to minimise complications, improve quality of life, and ensure timely intervention in cases of malignant transformation.
